# Unaccompanied Asylum-Seeking Refugee Children’s Forced Repatriation: *Social Workers’ and Police Officers’ Health and Job Characteristics*

**DOI:** 10.5539/gjhs.v7n6p215

**Published:** 2015-04-16

**Authors:** Johanna Sundqvist, Jonas Hansson, Mehdi Ghazinour, Kenneth Ögren, Mojgan Padyab

**Affiliations:** 1 Department of Public Health and Clinical Medicine, Epidemiology and Global Health, Umeå University, Sweden; 2 Basic Training Programme for Police Officers, Umeå University, Sweden; 3 Department of Social Work, Umeå University, Sweden

**Keywords:** general mental health, police officers, psychosocial job characteristics, social workers, unaccompanied asylum-seeking refugee children

## Abstract

During the past ten years the number of unaccompanied asylum-seeking refugee children has dramatically increased in Sweden. Some of them are permitted to stay in the receiving country, but some are forced back to their country of origin. Social workers and police officers are involved in these forced repatriations, and such complex situations may cause stressful working conditions. This study aimed to bridge the gap in knowledge of the relationship between general mental health and working with unaccompanied asylum-seeking refugee children who are due for forced repatriation. In addition, the role of psychosocial job characteristics in such relationships was investigated. A questionnaire including sociodemographic characteristics, the Swedish Demand-Control-Support Questionnaire, and the 12-item General Mental Health Questionnaire were distributed nationally. Univariate and multivariable regression models were used. Poorer mental health was associated with working with unaccompanied asylum-seeking refugee children among social workers but not among police officers. Psychological job demand was a significant predictor for general mental health among social workers, while psychological job demand, decision latitude, and marital status were predictors among police officers. Findings are discussed with special regard to the context of social work and police professions in Sweden.

## 1. Introduction

During the past ten years the number of refugee children and young people under the age of 18 who arrive on their own in Sweden to seek asylum without the care or support of parents or other relatives has increased more than 15 times (Swedish Migration Board, 2015). The Swedish Aliens Act (SFS 2005:716) states that these unaccompanied asylum-seeking refugee children, who are not considered to have needs for protection or other reasons to stay in Sweden, shall have the opportunity for a dignified repatriation. This can be to their country of origin, a transit country, or to another country where the child concerned voluntarily decides to go and in which he or she will be received (SFS 2005:716). The repatriation can occur voluntarily, be the result of complying with an obligation to return, or be enforced. In 2014, 26% of unaccompanied asylum-seeking refuge children’s asylum applications were rejected (Swedish Migration Board, 2015). As more children are seeking asylum, the number of rejections also increases. Both in literature and European Union (EU) guidelines, unaccompanied asylum-seeking refugee children are seen as a vulnerable group (2008/115/EC; Kohli, 2006). In forced repatriations, i.e., when the child refuses to leave the country voluntarily, social workers and police officers are key actors and their assignment from the Swedish government is to create both an efficient and dignified repatriation (SFS 2005:716; Swedish Government, 2014). In this process, social workers have a supportive role and are focused on the children’s mental health while the police officers have an executive role in the assignment to force the child to leave the country (Ghazinour et al., 2014).

Apart from social workers’ and police officers’ different roles, there are a number of structural differences between these two professions. For example, in Sweden police training is a vocational training, while social work training is an academic programme. Also, police officers operate within a government agency while social workers work in the municipally, indicating differences in organization and management. Additionally, the majority of police officers are men, while the majority of social workers are women. Furthermore, external guidance is not the practice in the police services, while it is customary among social workers.

Despite the differences in organizations and work tasks, both social work (Evans et al., 2006; Evans et al., 2005; Kahn, 1993; Padyab, Chelak, Nygren & Ghazinour, 2012; Tham & Meagher, 2009) and police work (Backteman-Erlanson, 2013; Backteman-Erlanson, Padyab, & Brulin, 2013; Chopko, 2010; Garbarino et al., 2011; Morash, Haarr & Kwak, 2006; Stinchcomb, 2004) are considered risky occupations for work stress. Johnson et al. (2005) compared the degree of work stress between 26 different occupations and found that two (out of six) of the identified occupations reporting worse-than-average stress evaluations were as social workers and police officers.

Karasek and Theorell (1990) described how work-related stress has an impact on health and identified two crucial psychosocial job characteristics that may impact the employee’s health, explicitly psychological job demands and control. According to Karasek and Theorell (1990), the psychological demands constitutes both the volume and intensity of one’s workload as well as how one copes with unforeseen tasks, while job control refers to the working individual’s potential control over the pace and content of his/her tasks. Control is further subdivided into skill discretion and decision authority but in the model this is combined into one single measure: decision latitude. Skill discretion is the breadth of skills workers can use on the job, while decision authority is related to control on the job, e.g. what tasks are to be performed, when, and in what order they will be done as well as when there will be a break. Control, or the decision latitude, can be close to or far from the working task. A close connection to the working task is control on the job, i.e., the performance of work, while a far connection is control over the job, i.e., the overall decisions. The latter is more related to a wider context than the immediate working environment, i.e., control on the job. Karasek and Theorell (1990) described how psychological demands and decision latitude affect strain, job satisfaction, and learning. Illness and mental stress occur when there is a disparity between perceived psychological job demands and workers’ control over the job situation.

Findings from a meta-analytic review showed that high levels of psychological demands and low decision latitude over the work situation was associated with a moderate risk of mental disorders, such as depression and anxiety (Stansfeld & Candy, 2006). The association between work stress and health outcomes, including mental health issues, has been investigated among police officers (Backteman-Erlanson, 2013; Chopko, 2010) and social workers (Evans et al., 2005; Peterson et al., 2008) separately.

However, a study of the two occupations would be beneficial to assess the magnitude of potential negative consequences of work stress on health in the two professions, especially in relation to forced repatriation of unaccompanied asylum-seeking refugee children. Dealing with repatriation of unaccompanied asylum-seeking refugee children means having to cope with children who have a fear of being repatriated or in other words dealing with children who are afraid of what will happen when they have returned to family or relatives. Sending such a child forcefully back to his/her home from a country he/she regarded as a safe haven may evoke stress for the professionals, thus leading to poorer mental health.

### 1.1 Aim

The aim of the study was to bridge the gap in knowledge on the relationship between general mental health and working with unaccompanied asylum-seeking refugee children who are due for repatriation among social workers and police officers. This subject is of high relevance because both professions work with the same target group and have to collaborate in the repatriation process. As one of the first studies on this topic, the present study aimed to (1) compare general mental health between those who had experiences with unaccompanied asylum-seeking refugee children’s forced repatriation and those without such experiences among social workers and police officers; (2) uncover the moderator effect of psychosocial job characteristics on such associations; and (3) explore similarities and differences of such associations in the two professions separately.

## 2. Methods

The research proposal was approved by the Regional Ethical Review Board at Umeå University, Dn 2014/69-31Ö.

### 2.1 Setting

#### 2.1.1 Social Services at the Municipality Level

Sweden consists of 290 municipalities, all of which have their own social services. The social services are an administration governed by separate municipal social welfare committees, which are responsible for the practical and political work regulated by the Social Services Act (SFS 2001:453). According to the Social Services Act, all municipalities have the ultimate responsibility of providing support to all individuals in need, including unaccompanied asylum-seeking refugee children for as long as they are present in the country. The social services can be organized in different ways, but all municipalities have statutory social workers responsible for unaccompanied asylum-seeking refuge children.

In the beginning of the asylum process, the social workers’ assignment is to investigate the children’s needs. This means, among other things, making decisions about interventions and accommodation (mostly care homes), appointing a legal guardian, and ensuring that the children receive schooling. Throughout the asylum process the social workers follow up on unaccompanied asylum-seeking refugee children’s mental health and living conditions through meetings with the children. In the repatriation process, social workers are responsible for the unaccompanied asylum-seeking refugee children’s mental health as well as to be a support, not only for the children but also for legal guardians and personnel at the care homes who have worked closest with the children.

#### 2.1.2 The Swedish Police Force

The Swedish Police Force was, through 2014, composed of 21 police county authorities (but from January 1, 2015, it was reorganized into one National Police Authority). Each county has a police authority responsible for daily police activities, with a chief commissioner as department head and a police board with politicians appointed by the government. In general, every police authority consists of an investigation and legal unit, a crime prevention unit, and a service unit. Most of the police authorities also have a border police unit. One specific task, often administrated by the border police unit, is to collaborate with the Swedish Migration Board (SMB) on unaccompanied asylum-seeking refugee children. The SMB is responsible for receiving and reviewing applications for asylum and, if it results in a negative asylum decision, managing the repatriation process all the way back to the country of origin. Where the child withholds or gives signals about not intending to contribute to the repatriation, the SMB submits the enforcement case to the police (SFS 2005:716). Contrary to the SMB, the police ultimately have the right to use methods to force the child to leave the country. When the police are responsible for the forced repatriation, they either travel on their own with the child to the home country or together with the Swedish Prison and Probation Service.

### 2.2 Sample and Data Collection

The current study was based on two national self-administered surveys, one of social workers and one of police officers, which were conducted in spring and autumn 2014.

#### 2.2.1 The Sample and Data Collection from Social Workers

All municipalities in Sweden in 2013 that had an agreement with the SMB on the reception of unaccompanied asylum-seeking refugee children were included in the study. This resulted in 265 municipalities. An e-mail list was established of all the social workers that work with unaccompanied asylum-seeking refugee children with or without a residence permit. Due to organizational factors, e-mail was the best way to get in touch with the social workers. Some e-mail addresses for social workers could be obtained from the country’s various county administration boards; these were sent to the research team. In cases where a county administration board had no indication of any municipalities or persons in the municipalities who worked with these issues, the research team contacted the municipality to get the names of the social workers. This was done in the form of an e-mail with an information letter or phone call. Once all the information was collected, the social workers were contacted to complete a Web-based survey.

A total of 506 social workers working with unaccompanied asylum-seeking refugee children received the Web-based questionnaire; of these, 133 replied. Two reminder e-mails went out two weeks apart. Those who were not involved with unaccompanied asylum-seeking refugee children were recruited from 247 social workers working with children and/or families in a municipality. The working tasks of these social workers were similar to the social workers who were working with unaccompanied asylum-seeking refugee children, but they were specifically working with other vulnerable children.

#### 2.2.2 The Sample and Data Collection From Police Officers

All 21 police authorities in Sweden were contacted. The project was described in a letter and through conversations with contacts that one of the researchers had with the police authorities. Three of the police authorities declined to participate in the study. Other police authorities received the survey from their human resource personnel unit or squad leaders. A paper survey was used due to technical factors concerning use of the Internet. The surveys and prepaid return envelopes were sent in a sealed envelope to the contact person at each authority who distributed them to the police officers that were able to respond to the survey and return it in the prepaid envelope. A total of 714 police officers responded and returned the questionnaires. This convenience sample was used due to secrecy rules in the Swedish police organization. Experience of working with unaccompanied asylum-seeking refugee children was identified using a single item question in the sociodemographic questionnaire (yes/no).

### 2.3 Instruments

The survey included an introductory letter stating the purpose of the study and a consent form, and a self-administered questionnaire was used to collect information on sociodemographic characteristics, psychosocial job characteristics, and general mental health of the participants.

Sociodemographic characteristics included age, gender, working experience, education, and marital status. Working experience referred to the number of years participants had been working in the profession. Education was a two-category variable: upper secondary education and higher education. Marital status was categorized into two groups: married/cohabiting and single. The latter included separated/divorced and widower/widow.

Psychosocial job characteristics were measured by the Swedish version of the Karasek demand/control model (Karasek & Theorell, 1990). This model is commonly used in occupational research and has been tested for reliability in the Swedish population (Theorell et al., 1988). Psychological job demands and decision latitude were calculated based on four psychological demands items and six decision latitude items on a four-point Likert scale, giving the score values 4–16 for psychological demands and 6–24 for decision latitude. Psychological demands and decision latitude were dichotomized by the median score.

The general health questionnaire (GHQ-12) was used as a self-administered screening test to assess general mental health. The GHQ-12 is a 12-item questionnaire developed to detect psychiatric disorders in community settings and non-psychiatric clinical settings (Arnetz, Arble, Backman, Lynch & Lublin, 2013; Banks et al., 1980; Goldberg & Williams, 1988). The psychometric properties of GHQ-12 have been extensively investigated. We have used the original scoring by Goldberg with response categories scored “not at all” and “no more than usual” as “0” and “rather more than usual” and “much more than usual” as “1”, giving a possible range from 0 to 12, with higher scores being indications of poorer mental health. In accordance with Goldberg, Oldehinkel, and Ormel’s (1998) recommendation “if the mean is below 1.85 then the threshold of 1/2, from 1.85 to 2.7 a threshold of 2/3, and above 2.7 a threshold of 3/4 seems to work best for the GHQ-12,” we decided to use the appropriate scores as the cut-off for the differentiation between individuals with and without psychological disturbance.

### 2.4 Statistical Methods

Pearson’s correlation coefficient was used to assess the association between general mental health and age, and between psychological demands and decision latitude total scores.

The chi-square test was used to compare the nominal variables and T-tests or the Mann-Whitney U test (depending on the Gaussian distribution) were used to compare continuous variables between males and females as well as between those with and without experience working with unaccompanied asylum-seeking refugee children. We applied univariate and multivariable logistic regression to describe the impact of demographic and psychosocial job characteristics on the likelihood of having psychological disturbance according to GHQ cut-off scores (psychological disturbance “yes or no”).

Statistical analyses were performed using the Statistical Package for the Social Sciences, version 22.0, software package (SPSS, Inc., Chicago, IL). Significance was established at p < 0.05.

## 3. Results

### 3.1 Social Workers

There were a total of 380 children and/or family social workers, consisting of 133 social workers who work specifically with unaccompanied asylum-seeking refugee children and 247 who were statutory social workers working with children and/or families in a municipality. The majority (95%) had a bachelor of science in social work while 5% had postgraduate degrees. The characteristics of the participants are presented in Table 1.

**Table 1:** Characteristics of the study participants, by gender

	Police Officers			Social Workers		
	
	Males	Females	Total	Males	Females	Total
N	59	321	380	494	220	714

Age Mean ± SD^a^ years	49 ± 12^**^	45 ± 11	45 ± 11	43 ± 11	39 ± 11^**^	42 ± 11

Range in Years	27-69	22-66	22-69	24-67	24-65	24-67

Marital Status % Married	85	74	76	87^**^	74	83

Work Experience Mean ± SD years	16 ± 11	15 ± 10	15 ± 10	16.7 ± 13.6^**^	12.6 ± 11.8	15.5 ± 13.3

Children % Yes	73	80	78	81^**^	65	76

Psychosocial Job Characteristics						

Psychological Demands at Scale 4-16 Mean ± SD	11.2 ± 2.5^**^	12.3 ± 2.5	12.1 ± 2.6	10.6 ± 2.0^*^	11 ± 2.1	10.7 ± 2.0

Decision Latitude at Scale 6-24 Mean ± SD	19.6 ± 1.9	19.8 ± 1.7	19.8 ± 1.7	19.2 ± 2.1	19.1 ± 1.8	19.2 ± 2.1

Note.

* p < 0.05

** p < 0.01 compared to females;

a SD = standard deviation.

The mean GHQ score was 3.11 among all social workers. Following the recommendations on cut-offs of GHQ, we decided to use a score of 4 (individuals with a total GHQ-12 score > 4 were considered as cases of psychological disturbance). According to the chosen cut-off, 38% of the social workers were regarded as possibly suffering from a psychological disturbance.

Mean GHQ scores were higher among social workers who worked with unaccompanied asylum-seeking refugee children, suggesting poorer general mental health compared to their counterparts (3.5 ± 3.3 vs. 2.8 ± 3.5, p < 0.01, Table 2).

**Table 2:** Mean and standard deviation of the GHQ total score by socio-demographic and psychosocial job characteristics of study participants

	Social Workers	p-value ^a^	Police Officers	p-value ^a^
Marital Status				
Married	2.9 ± 3.2	0.022	1.4 ± 2.3	0.002
Single	3.9 ± 3.8		2.1 ± 2.7	

Gender				
Male	2.1 ± 2.7	0.028	1.5 ± 2.4	0.077
Female	3.3 ± 3.5		1.7 ± 2.5	

Experience with UARC^8^				
Yes	3.5 ± 3.3	0.01	2.0 ± 2.9	0.2
No	2.8 ± 3.5		1.4 ± 2.2	

Psychosocial Job Characteristics				

Psychological Demands				
High	4.1 ± 3.5	0.001	2.1 ± 2.8	0.001
Low	1.6 ± 2.6		0.91 ± 1.7	

Decision Latitude				
Low	3.8 ± 3.5	0.001	1.8 ± 2.7	0.03
High	2.6 ± 3.2		1.2 ± 2.01	

Note.

^a^P-value obtained using Mann-Whitney test;

* Unaccompanied asylum-seeking refugee children

Those who had experience with unaccompanied asylum-seeking refugee children, recognize their job as providing higher psychological demands and lower decision latitude. Social workers with experience with unaccompanied asylum-seeking refugee children scored significantly higher on psychological demands compared to those who were not involved with unaccompanied asylum-seeking refugee children (12.8 ± 2.5 vs. 11.8 ± 2.5, respectively, p < 0.001). A similar pattern was found regarding job decision latitude. Social workers working with unaccompanied asylum-seeking refugee children scored lower on job decision latitude, suggesting lower control at work compared to their counterparts (19.4 ± 1.6 vs. 20 ± 1.7, respectively, p < 0.001). The calculated effect size for the difference for psychological demands and decision latitude between the two groups was 0.032 and 0.026 respectively. These effect sizes are considered as medium effect sizes (Tabachnick, 2006).

The GHQ was positively correlated with psychological demands, meaning higher demand was related to poorer mental health. The mean GHQ score was 4.1 ± 3.5 among those with high demand and 1.6 ± 2.6 among low-demand social workers (p < 0.001, Table 2).

The GHQ was negatively associated with decision latitude, meaning low decision latitude was correlated with poorer mental health. The mean GHQ was 3.8 ± 3.5 among those with high decision latitude and 2.6 ± 3.2 among low decision latitude social workers (p < 0.001, Table 2).

Pearson’s correlation coefficient for the association between general mental health, and psychological demands and decision latitude was 0.44 (p < 0.001) and -0.20 (p < 0.05) respectively suggesting a significant correlation between poorer mental health and high psychological demands, and low decision latitude (Table 3).

**Table 3:** Pearson’s correlation coefficient for the association between general mental health and age, psychological demands and decision latitude among social workers and police officers

Predictors	Social Workers	Police Officers
Age	-0.12 *	-0.06
Psychological Demands	0.44 ***	0.28 ***
Decision Latitude	-0.20 *	-0.20 ***

Note.

* p < 0.05

*** p < 0.001.

The results from the univariate logistic regression showed a 47% increase in odds of psychological disturbance for each unit increased in psychological demand (OR=1.47, 95% CI: 1.32-1.63, p < 0.001, Table 4), and a 19% decrease in psychological disturbance for each unit increased in decision latitude (OR=0.81, 95% CI: 0.71-0.91, p < 0.001, Table 4). The results from the multivariable logistic regression showed that only psychological demands was a significant predictor for psychological disturbances among social workers after controlling for age, gender, marital status, decision latitude, and experience of working with unaccompanied asylum-seeking refugee children (Table 4). This confirms that working with unaccompanied asylum-seeking refugee children was no longer a predictor for poorer general mental health after adjusting for psychosocial job characteristics.

**Table 4:** Logistic regression model for psychological disturbance among social workers and police officers

Predictors	Social Workers		Police Officers	

	Crude OR^a^ (95% CI ^b^)	Adjusted OR (95% CI)	Crude OR (95% CI)	Adjusted OR (95% CI)
Age ^c^	0.98 (0.96-0.99)*	0.99 (0.97-1.02)	0.98 (0.97-1.01)	0.99 (0.98-1.01)
Gender (ref=male)	1.60 (0.88-2.96)	1.10 (0.53-2.19)	1.36 (0.96-1.93)	1.13 (0.77-1.65)
Marital Status (ref=married)	1.57 (0.97-2.53)	1.50 (0.88-2.58)	2.01 (1.33-3.03)***	1.90 (1.24-3.02)**
Psychological Demands ^c^	1.47 (1.32-1.63)***	1.40 (1.25-1.56)***	1.36 (1.25-1.49)***	1.35 (1.23-1.48)***
Decision Latitude ^c^	0.81 (0.71-0.91)***	0.91 (0.78-1.06)	0.85 (0.79-0.92)***	0.86 (0.79-0.94)***
Experience UARC (ref=no)	1.54 (0.97-2.46)	0.98 (0.56-1.73)	1.31 (0.89-1.94)	1.21 (0.79-1.85)

Note.

a OR = odds ratio

b CI = confidence interval

c Age, psychological demands, and decision latitude are continuous variables with no reference group.

* p < 0.05

** p < 0.01

*** p < 0.001.

### 3.2 Police Officers

A total of 714 police officers participated in our study. There were 157 police officers (22%) who had experience working with unaccompanied asylum-seeking refugee children who were due for repatriation, 24% among males and 18% among females (Table 1).

Mean GHQ total score was 1.5 among police officers, with the given cut-off of 2 as possibly indicating psychological disturbance. According to the chosen cut-off, 28.5% of the police officers possibly suffered from a psychological disturbance.

Mean GHQ was not statistically different between those with and without experience with unaccompanied asylum-seeking refugee children (2.0 ± 2.9 vs. 1.4 ± 2.2, respectively, p = 0.2, Table 2).

Psychological demand was not statistically different between police officers with experience with unaccompanied asylum-seeking refugee children compared to those who were not involved with these children (10.9 ± 2.2 vs. 10.7 ± 1.9, respectively p = 0.1). The same pattern was found regarding job decision latitude; no difference was found between police officers with experience working with unaccompanied asylum-seeking refugee children compared to those without such experience (19.1 ± 2.4 vs. 19.2 ± 1.9, respectively, p = 0.6).

GHQ was positively correlated with psychological demands, meaning higher demand was related to poorer mental health. The mean GHQ was 2.1 ± 2.8 among those with high demand and 0.91 ± 1.7 among low-demand police officers (p < 0.001, Table 2).

GHQ was negatively associated with decision latitude, where low decision latitude was correlated with poorer mental health. The mean GHQ was 1.2 ± 2.01 among those with high decision latitude and 1.8 ± 2.7 among low decision latitude police officers (p = 0.03, Table 2).

Pearson’s correlation coefficient for the association between general mental health, and psychological demands and decision latitude was 0.28 and -0.20 (p < 0.001) respectively among police officers suggesting a significant correlation between poorer mental health and high psychological demands, and low decision latitude (Table 3).

The results from the univariate logistic regression showed that odds of psychological disturbance was 2.01 times higher for single individuals compare to married individuals (OR=2.01, 95% CI: 1.33-3.03, p < 0.001). A 36% increase in odds of psychological disturbance was found for each unit increase in psychological demands (OR=1.36, 95% CI: 1.25-1.49, p < 0.001), and a 15% decrease in odds of psychological disturbance for each unit increase in decision latitude (OR=0.85, 95% CI: 0.79-0.92, p < 0.001, Table 4). The results from the multivariable logistic regression showed that psychological demands, decision latitude, and marital status were still significant predictors for psychological disturbances among police officers after controlling for age, gender, and work with unaccompanied asylum-seeking refugee children (Table 4).

## 4. Discussion

The aim of the present study was to examine the general mental health of social workers and police officers with and without experience working with unaccompanied asylum-seeking refugee children, and whether these associations were moderated by psychosocial job characteristics. Further, we wanted to explore the similarities and differences between such associations in the two professions separately.

In summary, the results of this study suggest three key study findings based on the three research questions. First, significant for poorer general mental health was associated with working with unaccompanied asylum-seeking refugee children among social workers but not among police officers. Second, social workers perceived working with unaccompanied asylum-seeking refugee children as more stressful (indicated by higher psychological demands and lower decision latitude) than other social work. We could not find such an association among police officers. Third, considering all independent variables in the multivariate model, we found psychological demands was a significant predictor for psychological disturbance among social workers while psychological demands, decision latitude, and marital status were predictors among police officers.

### 4.1 Social Workers

In this study we have shown that the proportion of social workers with psychological disturbance was 38%. This could be compared with Tham’s (2009) study, which reported 31% of social workers had psychological disturbance. Moreover, our first key finding was that the social workers who were working with unaccompanied asylum-seeking refugee children were at higher risk for poorer mental health than the social workers who were not working with such children. As Chase (2010) described, social workers within the asylum system for unaccompanied asylum-seeking refuge children have an especially psychologically demanding work task to manage. They are expected to apply social care principles such as the best interest of the child, while they must also work within clear organizational boundaries with, for example, limited job resources. The fact that increasing numbers of unaccompanied asylum-seeking refugee children are coming to Sweden to seek asylum (Swedish Migration Board, 2015) sets high demands on social workers to be effective and make the right decisions in their job in order to give the children the support they need. The social workers also have to face the fact that the children are often forced to leave the country. According to interviewed social workers in our ongoing study (Ghazinour et al., 2014), this is an extremely demanding working situation where the social workers have to cope with unforeseen tasks and the feeling of lack of control over the forced repatriation process, as the police are the ones responsible for that latter process. This is in line with our second finding, that social workers perceived working with unaccompanied asylum-seeking refugee children as more stressful (indicated by higher psychological demands and lower decision latitude) than social workers working with other vulnerable children. The findings confirm not only the statistical significance, but also the magnitude of the mean difference of psychological demands and decision latitude between the two groups indicated by the medium effect size. In addition, we found approximately one unit difference in psychological demands and 0.6 for decision latitude between the two groups, which has practical significance. Maslach (1993) stressed that the relationship with the client is one of the most important tasks in social work, and thus the sensitivity to a client’s problems makes social workers vulnerable to work stress. We have seen that, depending on which role in the forced repatriation process professionals have (supportive or executive), they have different perceptions of what is best for the child (Ghazinour et al., 2014). Social workers who were identified as having a supportive role tended to believe that the best outcome for the unaccompanied asylum-seeking refugee child was to stay in Sweden and that forced repatriation could never be dignified. This is an incompatible goal, as the child de facto is going to be forced to leave the country. This impossible mission for the social workers—wanting to change the law or the outcome for the child—could be seen as not having “control over the job” (Karasek & Theorell, 1990). This could be one explanation for why social workers dealing with unaccompanied asylum-seeking refugee children perceived higher psychological demands and lower decision latitude than social workers working with other vulnerable children.

However, our third key finding, when we controlled for all independent variables, only psychological demands was a significant predictor for psychological disturbance among social workers. Considering the positive correlation between high psychological demands and poorer mental health, this means that the impact of working with unaccompanied asylum-seeking refugee children was explained by the already high demands when psychological demands as an explanatory variable was controlled for.

### 4.2 Police Officers

The findings of our study did not support the view that working with the forced repatriation of unaccompanied asylum-seeking refugee children negatively affected police officers’ general mental health. According to Karasek and Theorell (1990), psychological demands and control may impact an employee’s stress and health. High psychological demands and low decision latitude may have a negative effect on health. The police officers with experience of the forced repatriation of unaccompanied asylum-seeking refugee children did not perceive psychological demands or decision latitude differently compared to officers without such experience. Consequently, these job characteristics did not have a negative effect on their health. This may be related to their already demanding working tasks, which often expose the police officers to extreme situations (Paton & Smith, 1999). These experiences may make them accustomed to difficult situations.

According to Ghazinour et al. (2014), police officers tended to believe that a reunion with relatives in the country of origin was best for the child. This is consistent with their executive role in the forced repatriation work. As police work is to some extent corrective, police officers might be more task oriented; someone in society has to solve the problems, and this happens to be the police. Hence, police officers focus on their working task and solve a problem without considering it excessively. This may be a strategy to cope with demanding situations without being overly mentally affected. Furthermore, the forced repatriation task and the executive role require problem-solving police officers, and if the officers have what Karasek and Theorell (1990) call “skill discretion”, they would score higher on decision latitude. A problem-solving executive role could be a possible explanation for why police officers with experience of forced repatriation work did not perceive psychological demands or decision latitude differently compared to officers without such experience.

It was found that highly demanding working tasks related to poorer general mental health. This is in accordance with Karasek and Theorell (1990), Backteman-Erlanson, Padyab and Brulin (2013), and Chopko (2010), although Backteman and colleagues focused on burnout and Chopko’s focus was on post-traumatic distress. In addition, our findings were similar to those of Karasek and Theorell (1990), that poorer mental health was related to low decision latitude at work. Like the special unit police officers in Garbarino et al.’s (2011) study, the police officers were not mentally affected if they could control the situation, e.g., relevant training and appropriate organization of an event. Garbarino et al. (2011) have explored the relationship between the work context (routine work or special event) of special force policemen and psychological measures of job strain (demand–control) and effort–reward imbalance. They found that, in special police forces, routine work might be significantly more stressful than a single critical event. Another study related to this, Garbarino et al. (2012), found that police officers in a special force unit had a good capacity to withstand stress. In another study Garbarino, Chiorri, and Magnavita (2014), found that personality aspects may increase or mitigate the strain evoked by environmental stressors in special force units. In a multilevel study, Morash, Haarr and Kwak (2006) found no association between high crime rate and work stress; however, they emphasized that lack of influence over work activities was one important predictor of stress.

Finally, our third key finding when we controlled for all independent variables, was that psychological demands, decision latitude and marital status were significant predictors for general mental health among police officers. In other words poorer mental health was affected by high psychological demands, low decision latitude and being single.

### 4.3 Limitations

In cross-sectional studies no causality can be inferred, however important knowledge about the associations between different variables such as experience with unaccompanied asylum-seeking refugee children, psychological job demands, decision latitude, and the resulting GHQ can still be reported. The results might have been different if there had been a higher response rate among social workers, and the same applies to the convenience sample of police officers. Nevertheless, this is an association study and not a descriptive prevalence study, thus the bias may be a minor problem. Another limitation was the self-reported questionnaires. This procedure is not without bias, such as misunderstanding and misinterpretation from the respondents’ point of view. The aim of this study was not to make a direct statistical comparison between social workers and police officers. Future studies with particular focus on the comparison might be of importance to understand each other’s roles in working with vulnerable people.

## 5. Conclusion and Recommendations

There are several possible explanations for why social workers perceived working with unaccompanied asylum-seeking refugee children as more stressful than working with other vulnerable children, and why they had poorer general mental health. Some explanations might be related to their basic training, organization, management, or overall gender composition. One explanation might be their interpretation of their supportive role, which is incompatible with the forced repatriation process and therefore makes the psychological demands particularly high. This association was not seen for the police officers. They seemed not to be mentally affected, and this may be because they perceive their executive role in the forced repatriation process the same as any other work task. This difference may be related to both social workers’ and police officers’ perceptions of their roles in the forced repatriation process, and that may affect how they perceive both their psychological job demands and their general mental health.

Conditions for both occupations should be created in order to meet psychological demands, especially for the social workers in forced repatriation work. In order to decrease the psychological demands, we suggest that social workers in repatriation work should be strengthened in their role so their supportive work task does not become an impossible mission. Furthermore, we noticed that social workers need to receive feedback from the police about what happens with a child after the police have taken over the responsibility for the forced repatriation. To reach such an “end of the story” might be one way of increasing the low decision latitude and decreasing the high psychological demands. We also suggest that the overall collaboration between social workers and police officers in the forced repatriation process should be more frequent and incorporate a common goal in order to make the repatriation as dignified as possible for the child.
